# Psychological impact of the COVID-19 pandemic on primary care workers: a cross-sectional study

**DOI:** 10.3399/BJGP.2021.0691

**Published:** 2022-04-20

**Authors:** Enric Aragonès, Isabel del Cura-González, Lucía Hernández-Rivas, Elena Polentinos-Castro, Maria Isabel Fernández-San-Martín, Juan A López-Rodríguez, Josep M Molina-Aragonés, Franco Amigo, Itxaso Alayo, Philippe Mortier, Montse Ferrer, Víctor Pérez-Solà, Gemma Vilagut, Jordi Alonso, the MINDCOVID-19 research group

**Affiliations:** Institut Universitari d’Investigació en Atenció Primària Jordi Gol, Barcelona.; Research Network on Health Services in Chronic Diseases, Madrid; Department of Medical Specialties and Public Health, Universidad Rey Juan Carlos, Madrid; Primary Care Research Unit (GAAP-SERMAS), Madrid Health Service, Madrid; Gregorio Marañón Health Research Institute, Madrid.; La Paz University Hospital, Madrid.; Research Network on Health Services in Chronic Diseases, Madrid; Department of Medical Specialties and Public Health, Universidad Rey Juan Carlos, Madrid; Primary Care Research Unit (GAAP-SERMAS), Madrid Health Service, Madrid; Gregorio Marañón Health Research Institute, Madrid.; Institut Universitari d’Investigació en Atenció Primària Jordi Gol, Barcelona.; Research Network on Health Services in Chronic Diseases, Madrid; Department of Medical Specialties and Public Health, Universidad Rey Juan Carlos, Madrid; Primary Care Research Unit (GAAP-SERMAS), Madrid Health Service, Madrid; Gregorio Marañón Health Research Institute, Madrid.; Institut Català de la Salut, Barcelona.; Health Services Research Unit, Institut Hospital del Mar d’Investigacions Mèdiques, Barcelona; CIBERESP, Madrid.; Health Services Research Unit, Institut Hospital del Mar d’Investigacions Mèdiques, Barcelona; CIBERESP, Madrid.; Health Services Research Unit, Institut Hospital del Mar d’Investigacions Mèdiques, Barcelona; CIBERESP, Madrid.; Health Services Research Unit, Institut Hospital del Mar d’Investigacions Mèdiques, Barcelona; CIBERESP, Madrid; Department of Experimental and Health Sciences, Pompeu Fabra University, Barcelona.; Department of Psychiatry and Legal Medicine, Autonomous University of Barcelona, Barcelona; CIBER Mental Health (CIBERSAM), Madrid; Institut de Neuropsiquiatria i Addiccions, Parc de Salut Mar, Barcelona.; Health Services Research Unit, Institut Hospital del Mar d’Investigacions Mèdiques, Barcelona; CIBERESP, Madrid.; Health Services Research Unit, Institut Hospital del Mar d’Investigacions Mèdiques, Barcelona; CIBERESP, Madrid; Department of Experimental and Health Sciences, Pompeu Fabra University, Barcelona.

**Keywords:** COVID-19 pandemic, cross-sectional study, health personnel, mental health, primary health care, psychological resilience

## Abstract

**Background:**

The COVID-19 pandemic has had a major impact on the mental health of healthcare workers, yet studies in primary care workers are scarce.

**Aim:**

To investigate the prevalence of and associated factors for psychological distress in primary care workers during the first COVID-19 outbreak.

**Design and setting:**

This was a multicentre, cross-sectional, web-based survey conducted in primary healthcare workers in Spain, between May and September 2020.

**Method:**

Healthcare workers were invited to complete a survey to evaluate sociodemographic and work-related characteristics, COVID-19 infection status, exposure to patients with COVID-19, and resilience (using the Connor–Davidson Resilience Scale), in addition to being screened for common mental disorders (depression, anxiety disorders, post-traumatic stress disorder, panic attacks, and substance use disorder). Positive screening for any of these disorders was analysed globally using the term ‘any current mental disorder’.

**Results:**

A total of 2928 primary care professionals participated in the survey. Of them, 43.7% (95% confidence interval [CI] = 41.9 to 45.4) tested positive for a current mental disorder. Female sex (odds ratio [OR] 1.61, 95% CI = 1.25 to 2.06), having previous mental disorders (OR 2.58, 95% CI = 2.15 to 3.10), greater occupational exposure to patients with COVID-19 (OR 2.63, 95% CI = 1.98 to 3.51), having children or dependents (OR 1.35, 95% CI = 1.04 to 1.76 and OR 1.59, 95% CI = 1.20 to 2.11, respectively), or having an administrative job (OR 2.24, 95% CI = 1.66 to 3.03) were associated with a higher risk of any current mental disorder. Personal resilience was shown to be a protective factor.

**Conclusion:**

Almost half of primary care workers showed significant psychological distress. Strategies to support the mental health of primary care workers are necessary, including designing psychological support and resilience-building interventions based on risk factors identified.

## INTRODUCTION

The outbreak of the COVID-19 pandemic in March 2020 saturated the capacity of the Spanish healthcare system and forced organisational changes at all levels of care to adapt to the changing conditions.^1^ There was an important and abrupt change in the working conditions of primary care staff to meet new requirements, with staff having to tolerate uncertainties, organisational shortcomings, and a shortage of protective equipment.^2^ In Spain, primary care was responsible for the screening and diagnosis of patients with COVID-19, non-hospital treatment of most of the patients with COVID-19, and, in the initial moments of the collapse of the healthcare system, even complex home care for patients with COVID-19. Many primary care professionals took on occupational relocations and new tasks, such as working in nursing homes, COVID-19-specific field hospitals, and also relocations to hospital services.^3^^,^^4^

Overload and changes in working conditions, facing new and unfamiliar situations, lack of resources, fear of contagion, or fear of infecting family members generated significant stress in healthcare professionals. An increase in the prevalence of depression, anxiety, post-traumatic stress, drugs use, burnout, and increased risk of suicide have been described.^5^^–^7 Importantly, the psychological distress affecting healthcare workers not only has an impact on their wellbeing, but also their professional performance, quality of care, and patient safety.^8^ On the other hand, a sense of professional and civic responsibility has emerged in healthcare professionals,^9^^,^^10^ and staff have shown resilience in the face of insecurity and difficulties.

**Table:** How this fits in

In the context of the COVID-19 pandemic, a psychological impact on healthcare workers has been described, although studies in non-hospital settings are scarce. This study found that a high proportion of primary care workers (43.7%) had a current mental disorder. Female sex, having a history of previous mental disorders, greater work exposure to patients with COVID-19, having children or dependents, and certain professional positions were associated with greater risk. Personal resilience was shown to be a protective factor. Preventive and support interventions for the mental health of primary care workers are required.

Despite the abundant literature on this subject, few studies have specifically analysed the situation in primary care,^11^^–^15 notwithstanding the repercussions for those working in these settings and the different characteristics and conditions compared with those reported in hospital settings. In addition, females constitute the largest group within healthcare professions and yet most studies on the psychological impact of the pandemic on healthcare workers rarely mention sex as a variable affecting the results and they have not provided disaggregated data.^16^ This study therefore analysed the psychological distress experienced by primary care workers in the context of the COVID-19 pandemic, including a sex-disaggregated analysis.

The aim was to investigate psychological distress in Spanish primary care workers during the first COVID-19 outbreak period. Specifically, this study aimed to:

- estimate the prevalence of psychological distress by sex;
- evaluate the associations between psychological distress and sociodemographic, occupational, and health characteristics by sex; and
- explore the role of resilience as a protective factor.

## METHOD

### Design, population, and sampling

A multicentre, cross-sectional, web-based self-reported survey was conducted of Spanish healthcare workers between May and September of 2020 as part of the MINDCOVID-19 project.^17^ All workers in each healthcare institution included were invited to participate using administrative email distribution lists (that is, census sampling) that generated invitations to participate in the study containing an anonymous link to access the survey. A detailed description of the methods and procedures can be found in a previous article.^6^ The present study analysed the data obtained from professionals in the primary care settings of five autonomous communities in Spain (the Basque Country, Catalonia, Madrid, Castile and León, and Valencian Community). The staff in Spanish primary care centres comprise family doctors, paediatricians, dentists, nurses, auxiliary nurses, midwives, social workers, administrative staff, and other personnel.^18^

### Measurements

#### Sociodemographic and occupational characteristics

The survey included personal characteristics such as sex, age, marital status, having dependent children, caring for an older person or someone with disabilities, and profession.

#### Mental disorders

The survey screened for the following mental disorders: major depressive disorder, evaluated with the eight-item Patient Health Questionnaire;^19^^,^^20^ generalised anxiety disorder, evaluated with the seven-item Generalized Anxiety Disorder scale;^21^^,^^22^ panic attacks, evaluated via an item from the World Mental Health-International College Student;^23^^,^^24^ post-traumatic stress disorder (PTSD), evaluated with the PTSD Checklist for DSM-5;^25^ and substance use disorder, evaluated via the CAGE-AID questionnaire.^26^^,^^27^

The main variable, the presence of psychological distress, was considered present when there was a current positive screening for any of the above-mentioned mental disorders.

Mental disorders before the onset of the COVID-19 outbreak were recorded using a self-reported checklist based on the Composite International Diagnostic Interview, including lifetime depressive disorder, bipolar disorder, anxiety disorder, panic attacks, alcohol and drug use disorders, and other mental disorders.^28^^,^^29^

#### COVID-19 exposure and infection status

Participants were questioned about having been infected with SARS-CoV-2 and whether or not admission to hospital was necessary. Additionally, the responders were asked if their close ones (partner, children, parents, other relatives, or close friends) had contracted COVID-19. Occupational exposure to patients with COVID-19 was assessed using a five-level Likert scale (ranging from none of the time to all of the time).

#### Resilience

The 10-item Connor–Davidson Resilience Scale (CD-RISC-10)^30^^,^^31^ is a self-administered questionnaire with items rated on a five-point Likert scale (from 0, completely disagree to 4, completely agree) so that higher total scores indicate greater resilience.

### Ethical considerations

Before accessing the survey content, participants were informed about the objectives and procedures of the study, and their explicit consent for participation was obtained. The study was registered at ClinicalTrials.gov (reference: NCT04556565). As psychological distress could be revealed in the survey, participants were offered a list of local resources for mental health care.

### Statistical analysis

Participants who completed all the mental health items were included in the analysis. Sociodemographic, occupational, and health characteristics were compared between responders with and without psychological distress (that is, participants with and without a positive screening for any current mental disorder). To explore resilience, these variables were compared between participants with a resilience score above and below the 25th percentile. Categorical variables were analysed using the *χ*^2^-test, and the Mann–Whitney *U*-test was used for continuous variables.

A multivariable logistic regression model was estimated to assess potential factors associated with any current mental disorder. As the psychological impact of the pandemic can vary over time, the analyses were adjusted by the month of the response to the survey. A sex-stratified analysis was also conducted.

Statistical analyses were conducted using Stata (version 14). Statistical significance was set at *P*<0.05.

## RESULTS

### Response

A total of 3089 primary care professionals participated in the survey. Of these, 155 were excluded because of missing data in the questionnaires regarding mental health and six because of a lack of information on sex. Finally, 2928 participants were included in the statistical analysis.

The survey response rate was 12.5% in the main study when including all healthcare settings. The value for the primary care setting alone could not be calculated because the censuses of some of the participating centres include both primary care and hospital professionals.

### Participant characteristics

Table 1 shows participant characteristics, COVID-19 exposure, and infection status, as well as lifetime mental disorders. Of the participating sample, 82.7% were female and the median age was 50 years (interquartile range 42–57). Most responders were physicians (47.9%), followed by nurses and auxiliary nurses (29.8%), and administrative staff (11.1%). Of all participants, 41.6% reported any lifetime mental disorder before the COVID-19 outbreak.

**Table 1.** Sociodemographic and occupational characteristics, COVID-19 exposure, infection status, and lifetime mental disorders in primary healthcare workers

**Characteristic**	**Total (*n* = 2928),^a^ *n* (%)**	**Male (*n* = 506),^a^ *n* (%)**	**Female (*n* = 2422),^a^ *n* (%)**	***P*-value^b^**
**Age, years**	2928	506	2422	<0.001
18–29	207 (7.1)	27 (5.3)	180 (7.4)	—
30–49	1185 (40.5)	166 (32.8)	1019 (42.1)	—
≥50	1536 (52.5)	313 (61.9)	1223 (50.5)	—

**Marital status^c^**	2923	505	2418	0.025
Single, divorced/separated, or widowed	1166 (39.9)	179 (35.4)	987 (40.8)	—
Married	1757 (60.1)	326 (64.6)	1431 (59.2)	—

**Children in care^c^**	2840	493	2347	0.068
Aged ≤12 years	752 (26.5)	110 (22.3)	642 (27.4)	—
Aged >12 years	516 (18.2)	93 (18.9)	423 (18.0)	—
None	1572 (55.4)	290 (58.8)	1282 (54.6)	—

**Caring for older person or person with disabilities**	2464	420	2044	0.003
Yes	336 (13.6)	38 (9.0)	298 (14.6)	
No	2128 (86.4)	382 (91.0)	1746 (85.4)	

**Profession**	2892	500	2392	<0.001
Physician	1384 (47.9)	298 (59.6)	1086 (45.4)	—
Nurse or auxiliary nurse	863 (29.8)	85 (17.0)	778 (32.5)	—
Administrative staff	322 (11.1)	54 (10.8)	268 (11.2)	—
Other staff involved in patient care	228 (7.9)	34 (6.8)	194 (8.1)	—
Other staff not involved in patient care	95 (3.3)	29 (5.8)	66 (2.8)	—

**Frequency of direct exposure to patients with COVID-19**	2846	496	2350	0.015
All/most of the time	1357 (47.7)	238 (48.0)	1119 (47.6)	—
Some of the time	1041 (36.6)	161 (32.5)	880 (37.4)	—
A little/none of the time	448 (15.7)	97 (19.6)	351 (14.9)	—

**Close one infected with COVID-19**	2926	506	2420	0.502
No	542 (18.5)	103 (20.4)	439 (18.1)	—
Close one infected, not family member	1721 (58.8)	292 (57.7)	1429 (59.0)	—
Family member infected	663 (22.7)	111 (21.9)	552 (22.8)	—

**COVID-19 infection status**	2923	505	2418	0.053
Admission to hospital	39 (1.3)	12 (2.4)	27 (1.1)	—
Test positive/diagnosed	548 (18.7)	101 (20.0)	447 (18.5)	—
None	2336 (79.9)	392 (77.6)	1944 (80.4)	—

**Resilience score, CD-RISC-10, median (IQR)^c^**	29.0 (25.0–33.0)	30.0 (26.0–35.0)	29.0 (24.0–33.0)	<0.001

**Lifetime mental disorders before COVID-19 outbreak**	2895	501	2394	0.070
Yes	1203 (41.6)	190 (37.9)	1013 (42.3)	
No	1692 (58.4)	311 (62.1)	1381 (57.7)	

a *Unless stated otherwise.*

b *Mann–Whitney* U*-test for continuous variables and χ^2^-test for categorical variables.*

c *Total,* n *= 2744; males,* n *= 485; and females,* n *= 2259. CD-RISC-10 = 10-item Connor–Davidson Resilience Scale. Close one = partner, children, parents, other relatives, or close friends. IQR = interquartile range.*

### Prevalence of any current mental disorder

The global prevalence of a positive screening for any current mental disorder was 43.7% (95% confidence interval [CI] = 41.9 to 45.4). The prevalence was significantly lower for males (33.8%, 95% CI = 29.7 to 37.9) than for females (45.7%, 95% CI = 43.7 to 47.7) (data not shown).

### Factors associated with any current mental disorder

Table 2 shows the associations between the characteristics of participants and a positive screening for any current mental disorder, stratified by sex. Statistically significant differences in age and profession were found. Caring for people was associated with a higher prevalence of a current mental disorder in females, but these differences were not significant among males. The presence of a lifetime mental health disorder was associated with a positive screening for any current mental disorder.

**Table 2.** Prevalence of positive screening for any current mental disorder according to the characteristics of primary care workers, disaggregated by sex

**Characteristic**	**Total (*n* = 1278) *n* (%)^a^**	***P*-value for *χ*^2^**	**Male (*n* = 171) *n* (%)^a^**	***P*-value for *χ*^2^**	**Female (*n* = 1107) *n* (%)^a^**	***P*-value for *χ*^2^**
**Age, years**		<0.001		0.003		<0.001
18–29	99 (47.8)	—	7 (25.9)	—	92 (51.1)	—
30–49	586 (49.5)	—	73 (44.0)	—	513 (50.3)	—
≥50	593 (38.6)	—	91 (29.1)	—	502 (41.0)	—

**Marital status**		0.04		0.085		0.18
Single, divorced/separated, or widow/er	537 (46.1)	—	69 (38.5)	—	468 (47.4)	—
Married	740 (42.1)	—	101 (31.0)	—	639 (44.7)	—

**Children in care**		0.02		0.628		0.03
Aged ≤12 years	362 (48.1)	—	40 (36.4)	—	322 (50.2)	—
Aged >12 years	222 (43.0)	—	28 (30.1)	—	194 (45.9)	—
None	659 (41.9)	—	100 (34.5)	—	559 (43.6)	—

**Caring for older person or person with disabilities**		0.003		0.62		0.009
Yes	170 (50.6)	—	13 (34.2)	—	157 (52.7)	—
No	894 (42.0)	—	116 (30.4)	—	778 (44.6)	—

**Profession**		<0.001		0.005		<0.001
Physician	544 (39.3)	—	84 (28.2)	—	460 (42.4)	—
Nurse or auxiliary nurse	403 (46.7)	—	34 (40.0)	—	369 (47.4)	—
Administrative staff	179 (55.6)	—	27 (50.0)	—	152 (56.7)	—
Other staff involved in patient care	90 (39.5)	—	11 (32.4)	—	79 (40.7)	—
Other staff NOT involving patient care	47 (49.5)	—	14 (48.3)	—	33 (50.0)	—

**Frequency of direct exposure to patients with COVID-19**		<0.001		0.33		<0.001
All/most of the time	665 (49.0)	—	88 (37.0)	—	577 (51.6)	—
Some of the time	436 (41.9)	—	48 (29.8)	—	388 (44.1)	—
A little/none of the time	145 (32.4)	—	33 (34.0)	—	112 (31.9)	—

**Close one infected with COVID-19**		0.35		0.47		0.12
No	241 (44.5)	—	30 (29.1)	—	211 (48.1)	—
Close one infected, not family member	763 (44.3)	—	100 (34.2)	—	663 (46.4)	—
Family member infected	273 (41.2)	—	41 (36.9)	—	232 (42.0)	—

**COVID-19 infection status**		0.06		0.18		0.14
Admission to hospital	24 (61.5)	—	7 (58.3)	—	17 (63.0)	—
Positive test/diagnosis	246 (44.9)	—	35 (34.7)	—	211 (47.2)	—
None	1007 (43.1)	—	129 (32.9)	—	878 (45.2)	—

**Lifetime mental disorders before COVID-19 outbreak**		<0.001		<0.001		<0.001
Yes	712 (59.2)	—	93 (48.9)	—	619 (61.1)	—
No	558 (33.0)	—	77 (24.8)	—	481 (34.8)	—

a *Percentages calculated from responders for each cell in Table 1. Close one = partner, children, parents, other relatives, or close friends.*

### Resilience

Resilience was associated with sex, profession, and lifetime mental health disorders (Table 3). Lower resilience was observed in females, administrative staff, responders with former mental health disorders, and those who declared being treated for such disorders.

**Table 3.** Associations of sociodemographic and job characteristics, and lifetime mental health disorders with resilience in primary healthcare workers

**Characteristic**	**Resilience score, CD-RISC-10**		

	**Under 25th percentile (*n* = 660), *n* (%)**	**Over 25th percentile (*n* = 2084), *n* (%)**	***P*-value^a^**
**Sex**			0.003
Male	91 (18.8)	394 (81.2)	—
Female	569 (25.2)	1690 (74.8)	—

**Age, years**			0.23
18–29	49 (25.8)	141 (74.2)	—
30–49	285 (25.5)	834 (74.5)	—
≥50	326 (22.7)	1109 (77.3)	—

**Marital status**			0.99
Single, divorced/separated or widowed	262 (24.1)	826 (75.9)	—
Married	398 (24.1)	1254 (75.9)	—

**Children in care**			0.298
Aged ≤12 years	167 (22.9)	563 (77.1)	—
Aged >12 years	111 (22.3)	387 (77.7)	—
None	382 (25.2)	1134 (74.8)	—

**Caring for older person or person with disabilities**			0.57
Yes	74 (22.9)	249 (77.1)	—
No	502 (24.4)	1559 (75.6)	—

**Profession**			0.008
Physician	313 (23.7)	1008 (76.3)	—
Nurse or auxiliary nurse	206 (25.4)	604 (74.6)	—
Administrative staff	87 (29.0)	213 (71.0)	—
Other profession involved in patient care	36 (16.4)	183 (83.6)	—
Other staff NOT involved in patient care	16 (18.0)	73 (82.0)	—

**Frequency of direct exposure to patients with COVID-19**			
All/most of the time	298 (22.8)	1007 (77.2)	0.34
Some of the time	253 (25.4)	743 (74.6)	—
A little/none of the time	109 (24.8)	331 (75.2)	—

**Close one infected with COVID-19**			0.05
No	142 (28.0)	366 (72.0)	
Close one infected, not family member	365 (22.6)	1249 (77.4)	
Family member infected	153 (24.7)	467 (75.3)	

**COVID-19 infection status**			0.10
Admission to hospital	11 (31.4)	24 (68.6)	—
Test positive/diagnosed	138 (27.1)	371 (72.9)	—
None	509 (23.2)	1686 (76.8)	—

**Lifetime mental disorders before COVID-19 outbreak**			<0.001
Yes	370 (32.6)	764 (67.4)	—
No	285 (18.0)	1296 (82.0)	—

a *Mann–Whitney* U*-test for continuous variables and χ^2^-test for categorical variables. CD-RISC-10 = 10-item Connor–Davidson Resilience Scale. Close one = partner, children, parents, other relatives, or close friends. IQR = interquartile range.*

### Models

Table 4 shows the multivariate analyses of the associations between any current mental disorder and the characteristics of the responders. Being aged 30–49 years, having children aged >12 years, caring for an older person or someone with disablities, being a nurse or auxiliary nurse, or administrative staff, and being exposed to patients with COVID-19 were associated with a higher risk of mental disorder, both for the complete sample and in females alone. However, these associations were not present in males. Having a history of any lifetime mental disorder was associated with a higher risk of a current mental disorder. Resilience was shown to be a protective factor for any current mental disorder.

**Table 4.** Multivariate associations between primary care workers’ characteristics and lifetime mental disorders, stratified by sex^a^

**Characteristic**	**Total (*n* = 2355), OR (95% CI)**	***P*-value**	**Male (*n* = 408), OR (95% CI)**	***P*-value**	**Female (*n* = 1947), OR (95% CI)**	***P*-value**
**Sex**						
Male	Reference	—	NA	—	NA	—
Female	1.61 (1.25 to 2.06)	<0.001	NA	—	NA	—

**Age, years**						
18–29	1.12 (0.75 to 1.66)	0.588	0.34 (0.09 to 1.35)	0.124	1.34 (0.87 to 2.05)	0.180
30–49	1.50 (1.19 to 1.88)	0.001	1.30 (0.72 to 2.33)	0.387	1.53 (1.19 to 1.97)	0.001
≥50	Reference	—	Reference	—	Reference	—

**Children in care**						
None	Reference	—	Reference	—	Reference	—
Aged ≤12 years	1.18 (0.91 to 1.51)	0.209	1.19 (0.63 to 2.23)	0.597	1.21 (0.92 to 1.60)	0.176
Aged >12 years	1.31 (1.03 to 1.67)	0.026	1.11 (0.60 to 2.03)	0.746	1.35 (1.04 to 1.76)	0.025

**Caring for older person or person with disabilities**	1.54 (1.18 to 2.00)	0.001	1.38 (0.62 to 3.06)	0.428	1.59 (1.20 to 2.11)	0.001

**Profession**						
Physician	Reference	—	Reference	—	Reference	—
Nurse or auxiliary nurse	1.34 (1.09 to 1.65)	0.006	1.49 (0.81 to 2.75)	0.204	1.33 (1.06 to 1.66)	0.012
Administrative staff	2.24 (1.66 to 3.03)	<0.001	1.69 (0.82 to 3.49)	0.157	2.39 (1.70 to 3.35)	<0.001
Other staff involved in patient care	1.08 (0.76 to 1.54)	0.660	1.18 (0.47 to 3.01)	0.723	1.09 (0.74 to 1.59)	0.668
Other staff not involved in patient care	2.22 (1.30 to 3.81)	0.004	2.24 (0.76 to 6.58)	0.142	2.09 (1.12 to 3.88)	0.020

**Frequency of direct exposure to patients with COVID-19**						
A little/none of the time	Reference	—	Reference	—	Reference	—
Some of the time	1.88 (1.40 to 2.52)	<0.001	1.15 (0.56 to 2.37)	0.712	2.06 (1.49 to 2.84)	<0.001
All/most of the time	2.63 (1.98 to 3.51)	<0.001	1.61 (0.80 to 3.22)	0.183	2.90 (2.11 to 3.99)	<0.001

**Resilience score, CD-RISC-10**	0.93 (0.92 to 0.95)	<0.001	0.91 (0.88 to 0.95)	<0.001	0.94 (0.92 to 0.95)	<0.001

**Any lifetime mental disorder**	2.58 (2.15 to 3.10)	<0.001	2.57 (1.60 to 4.12)	<0.001	2.59 (2.12 to 3.16)	<0.001

a *Exponentiated coefficients, adjusted by month of survey. Total model: pseudo-R^2^ 0.1174; AIC 2874.2; BIC 2966.4; and AUC 0.72. Male model: pseudo-R^2^ 0.1304; AIC 467.2; BIC 527.4; and AUC 0.74. Female model: pseudo-R^2^ 0.1090; AIC 2422.4; BIC 2506.0; and AUC 0.71. AIC = Akaike Information Criterion. AUC = area under the curve. BIC = Bayesian Information Criterion. CD-RISC-10 = 10-item Connor–Davidson Resilience Scale. NA = Not applicable. OR = odds ratio.*

## DISCUSSION

### Summary

The outcomes of the present study show that a high proportion (43.7%) of primary care workers screened positive for any current mental disorder; the proportion being significantly higher in females than in males. Female sex, having a previous history of mental disorders, greater occupational exposure to patients with COVID-19, caring for children or dependents, or certain occupations were factors that were independently associated with an increased risk of having a mental disorder, whereas resilience was shown to be a protective factor.

### Strengths and limitations

This study is particularly relevant because it evaluated the impact of the pandemic on primary care professionals, whose work characteristics and pandemic-related experiences differ greatly from those of hospital workers, the latter being more widely studied in the scientific literature.^32^ A strength of this study is that other professional profiles aside from doctors or nurses were included; previous studies have rarely included this data. This allowed confirmation of the significant psychological repercussions of the pandemic on administrative personnel.

When interpreting these results, it should be kept in mind that females represent 83% of the participants, which, far from constituting a bias, is a reflection of the reality of the healthcare work setting, where females are the vast majority in all professional categories in European health systems and, in particular, in the Spanish health system.^33^^,^^34^ One of the strengths of the present analysis lies in the reporting of sex-disaggregated data.

This study has several limitations. First, participation was voluntary, which may have introduced a difficult-to-predict bias because of self-selection of participants in the survey.^35^ This is especially important when the non-response rate is high, although this limitation is inherent to the methodology employed and is similar to other studies based on telematic surveys.^36^ Second, in a cross-sectional study, causality cannot be inferred from the factors associated with the assessed outcomes. Observing the evolution over time of psychological distress as a function of experience with the pandemic will be necessary to establish causal relationships. Indeed, this is precisely the objective of a prospective follow-up of this cohort currently underway.^6^ Third, the presence of probable mental disorders has been assessed by a battery of screening instruments. Establishing genuine clinical diagnoses was not possible, but positive screenings can be a valid indicator of the presence of significant psychological distress.^37^^,^^38^ Finally, when interpreting the data from this cross-sectional study, the time at which they were obtained, between the end of the first wave and the beginning of the second wave in the pandemic epidemiological curve in Spain, must be considered.^39^

### Comparison with existing literature

Differences in the prevalence of psychological distress by sex are to be expected, as a higher prevalence of mental disorders in females is a consistent finding in epidemiological studies.^40^^,^^41^ Greater vulnerability in females has also been reported among healthcare workers during the pandemic.^42^^–^44 Various explanations for these differences have been proposed, including response bias (males would have greater difficulty recognising and communicating psychological distress), as well as biological, social, and demographic factors.^45^^,^^46^ This study found that having children aged >12 years or caring for an older person or person with disabilities are important risk factors for psychological distress in females, whereas this association was not observed in males. This suggests that different family roles may be a key factor in sex-related differences in emotional distress.^16^^,^^47^ In addition, differences in informal caregiving between sexes may have increased following the shutdown of or limited access to resources such as childcare centres, schools, daycare nursing centres, or residences for older people.^48^ A qualitative study involving healthcare workers in England shows caring responsibilities as a factor that affects males and females differently in terms of their emotional state during the pandemic.^16^

As expected, the greater the occupational exposure to patients with COVID-19, the greater the risk of psychological distress for the overall sample; an association that is stronger and more consistent in females than in males.^49^^,^^50^ However, similar to findings from other research,^51^^–^53 this study found the paradox that administrative personnel were at greater risk than professional groups with direct patient contact. Again, these associations are strong and statistically significant in females, but not in males. As a result of the pandemic, primary care administrative staff have been exposed to changes, uncertainty, and a heavy workload, perhaps without sufficient support to handle this type of situation and with less control over their job conditions than other professional categories.^54^ In contrast, female doctors experienced less psychological distress than those in other occupations, possibly because of skills and experience in managing and coping with situations of complexity and uncertainty inherent to medical practice.^55^

The association between the existence of previous mental disorders and the current presence of any mental disorder was particularly strong, being comparable in both sexes. This was to be expected given the tendency for recurrence and the often chronic nature of mental disorders,^56^ and is consistent with other studies in healthcare workers in the pandemic setting.^44^^,^^57^ The relevance of this risk factor is accentuated by the fact that 42% of the individuals in the present sample reported a history of previous mental disorders.

Resilience is an individual’s ability to cope with and adapt to adverse situations while maintaining effective personal and professional functioning.^58^ Concurring with a study on healthcare workers in Italy,^59^ this work identified resilience as a protective factor against the psychological distress caused by the pandemic in healthcare professionals, both in males and females, although the level of resilience was higher among males.^60^ This ability to cope with stress was shown to be significantly impaired in those individuals with a previous history of mental disorders.

### Implications for research and practice

This study found that a high proportion of primary healthcare workers experienced psychological distress in the context of the COVID-19 pandemic and some particularly vulnerable profiles were identified. Given this situation, establishing strategies and interventions for psychological support and resilience building of healthcare workers is highly relevant, taking into account the risk factors identified and tailoring the interventions accordingly. Proactive systems should be established to assess and monitor the psychological wellbeing of different professional groups in primary care and facilitate their access to psychological help.^61^ Additionally, interventions should be conducted to promote resilience, as it is a modifiable factor,^62^^,^^63^ implementing strategies focused on self-care and changes in the organisation and work environment.^64^^,^^65^

Longitudinal studies are necessary to assess the evolution of the psychological impact of the pandemic over time and to identify the factors that determine or can predict this evolution. Evaluating the usefulness, feasibility, and effectiveness of any preventive or therapeutic interventions under real conditions will also be important, as well as determining the best way to implement them.^66^

## Data

The study database is available from the authors on reasonable request, following approval of a proposal and with a signed data-access agreement.

## References

[1] Legido-Quigley H, Mateos-García JT, Campos VR (2020). The resilience of the Spanish health system against the COVID-19 pandemic. Lancet Public Health.

[2] de Sutter A, Llor C, Maier M (2020). Family medicine in times of ‘COVID-19’: a generalists’ voice. Eur J Gen Pract.

[3] Muñoz MA, López-Grau M (2020). Lessons learned from the approach to the COVID-19 pandemic in urban primary health care centres in Barcelona, Spain. Eur J Gen Pract.

[4] Fernández-Aguilar C, Casado-Aranda LA, Farrés Fernández M, Minué Lorenzo S (2021). Has COVID-19 changed the workload for primary care physicians? The case of Spain. Fam Pract.

[5] Mortier P, Vilagut G, Ferrer M (2021). Thirty-day suicidal thoughts and behaviors among hospital workers during the first wave of the Spain COVID-19 outbreak. Depress Anxiety.

[6] Alonso J, Vilagut G, Mortier P (2021). Mental health impact of the first wave of COVID-19 pandemic on Spanish healthcare workers: a large cross-sectional survey. Rev Psiquiatr Salud Ment (Engl Ed).

[7] de Sousa GM, Tavares VDO, de Meiroz Grilo MLP (2021). Mental health in COVID-19 pandemic: a meta-review of prevalence meta-analyses. Front Psychol.

[8] Tawfik DS, Scheid A, Profit J (2019). Evidence relating health care provider burnout and quality of care: a systematic review and meta-analysis. Ann Intern Med.

[9] Harkin D (2021). COVID-19 and medical professionalism in a pandemic. Postgrad Med J.

[10] Goddard AF, Patel M (2021). The changing face of medical professionalism and the impact of COVID-19. Lancet.

[11] Zeng X, Peng T, Hao X (2021). Psychological distress reported by primary care physicians in china during the COVID-19 pandemic. Psychosom Med.

[12] Lasalvia A, Rigon G, Rugiu C (2021). The psychological impact of COVID-19 among primary care physicians in the province of Verona, Italy: a cross-sectional study during the first pandemic wave. Fam Pract.

[13] Amerio A, Bianchi D, Santi F (2020). Covid-19 pandemic impact on mental health: a web-based cross-sectional survey on a sample of Italian general practitioners. Acta Biomed.

[14] Lange M, Joo S, Couette PA (2022). Impact on mental health of the COVID-19 outbreak among general practitioners during the sanitary lockdown period. Ir J Med Sci.

[15] Lum A, Goh YL, Wong KS (2021). Impact of COVID-19 on the mental health of Singaporean GPs: a cross-sectional study. BJGP Open.

[16] Regenold N, Vindrola-Padros C (2021). Gender matters: a gender analysis of healthcare workers’ experiences during the first COVID-19 pandemic peak in England. Soc Sci.

[17] Alonso J, Martin JDDM, Ortí-Lucas RM MINDCOVID: mental health in a pandemic. https://studies.epidemixs.org/en/proyecto/mindcovid-study-covid-19-mental-health.

[18] Martí T, Peris A, Cerezo J (2021). Country vignette. Spain transforming primary health care during the pandemic: accelerating multidisciplinary teamwork to address emerging primary care needs in three Spanish regions.

[19] Wu Y, Levis B, Riehm KE, Saadat N (2020). Equivalency of the diagnostic accuracy of the PHQ-8 and PHQ-9: a systematic review and individual participant data meta-analysis. Psychol Med.

[20] Díez-Quevedo C, Rangil T, Sánchez-Planell L (2001). Validation and utility of the patient health questionnaire in diagnosing mental disorders in 1003 general hospital Spanish inpatients. Psychosom Med.

[21] Newman MG, Zuellig AR, Kachin KE (2002). Preliminary reliability and validity of the generalized anxiety disorder questionnaire-IV: a revised self-report diagnostic measure of generalized anxiety disorder. Behav Ther.

[22] García-Campayo J, Zamorano E, Ruiz MA (2010). Cultural adaptation into Spanish of the generalized anxiety disorder-7 (GAD-7) scale as a screening tool. Health Qual Life Outcomes.

[23] Kessler RC, Santiago PN, Colpe LJ (2013). Clinical reappraisal of the Composite International Diagnostic Interview Screening Scales (CIDI-SC) in the Army Study to Assess Risk and Resilience in Service members (Army STARRS). Int J Methods Psychiatr Res.

[24] Blasco MJ, Castellví P, Almenara J (2016). Predictive models for suicidal thoughts and behaviors among Spanish university students: rationale and methods of the UNIVERSAL (university & mental health) project. BMC Psychiatry.

[25] Zuromski KL, Ustun B, Hwang I (2019). Developing an optimal short-form of the PTSD Checklist for DSM-5 (PCL-5). Depress Anxiety.

[26] Mdege ND, Lang J (2011). Screening instruments for detecting illicit drug use/abuse that could be useful in general hospital wards: a systematic review. Addict Behav.

[27] Díez-Martínez S, Martín-Moros JM, Altisent-Trota R (1991). Brief questionnaires for the early detection of alcoholism in primary health care. Aten Primaria.

[28] Kessler RC, Ustun TB (2004). The World Mental Health (WMH) survey initiative version of the World Health Organization (WHO) Composite International Diagnostic Interview (CIDI). Int J Methods Psychiatr Res.

[29] Alonso J, Angermeyer MC, Bernert S (2004). Sampling and methods of the European Study of the Epidemiology of Mental Disorders (ESEMeD) project. Acta Psychiatr Scand Suppl.

[30] Connor KM, Davidson JRT (2003). Development of a new resilience scale: the Connor-Davidson Resilience Scale (CD-RISC). Depress Anxiety.

[31] Notario-Pacheco B, Solera-Martínez M, Serrano-Parra MD (2011). Reliability and validity of the Spanish version of the 10-item Connor-Davidson Resilience Scale (10-item CD-RISC) in young adults. Health Qual Life Outcomes.

[32] Sanghera J, Pattani N, Hashmi Y (2020). The impact of SARS-CoV-2 on the mental health of healthcare workers in a hospital setting — a systematic review. J Occup Health.

[33] Vázquez Vega P (2010). [The feminisation of health professionals]. [Article in Spanish]. https://www.fbbva.es/wp-content/uploads/2017/05/dat/DE_2010_feminizacion_profesiones_sanitarias.pdf.

[34] Eurostat (2021). Majority of health jobs held by women. https://ec.europa.eu/eurostat/web/products-eurostat-news/-/edn-20210308-1.

[35] Lin YH, Chen CY, Wu SI (2021). Efficiency and quality of data collection among public mental health surveys conducted during the COVID-19 pandemic: systematic review. J Med Internet Res.

[36] Cunningham CT, Quan H, Hemmelgarn B (2015). Exploring physician specialist response rates to web-based surveys. BMC Med Res Methodol.

[37] Kroenke K, Strine TW, Spitzer RL (2009). The PHQ-8 as a measure of current depression in the general population. J Affect Disord.

[38] Ruiz MA, Zamorano E, García-Campayo J (2011). Validity of the GAD-7 scale as an outcome measure of disability in patients with generalized anxiety disorders in primary care. J Affect Disord.

[39] Carlos III Health Institute (2022). [Covid in Spain. Situation and evolution of the COVID-19 pandemic in Spain. Epidemic curve of the pandemic]. https://cnecovid.isciii.es/covid19/#ccaa.

[40] Van de Velde S, Boyd A, Villagut G (2019). Gender differences in common mental disorders: a comparison of social risk factors across four European welfare regimes. Eur J Public Health.

[41] Maestre-Miquel C, López-de-Andrés A, Ji Z (2021). Gender differences in the prevalence of mental health, psychological distress and psychotropic medication consumption in Spain: a nationwide population-based study. Int J Environ Res Public Health.

[42] Liu S, Yang L, Zhang C (2021). Gender differences in mental health problems of healthcare workers during the coronavirus disease 2019 outbreak. J Psychiatr Res.

[43] Di Tella M, Romeo A, Benfante A, Castelli L (2020). Mental health of healthcare workers during the COVID-19 pandemic in Italy. J Eval Clin Pract.

[44] López-Atanes M, Pijoán-Zubizarreta JI, González-Briceño JP (2021). Gender-based analysis of the psychological impact of the COVID-19 pandemic on healthcare workers in Spain. Front Psychiatry.

[45] Klose M, Jacobi F (2004). Can gender differences in the prevalence of mental disorders be explained by sociodemographic factors?. Arch Womens Ment Health.

[46] Kuehner C (2017). Why is depression more common among women than among men?. Lancet Psychiatry.

[47] Bracke P, Christiaens W, Wauterickx N (2008). The pivotal role of women in informal care. J Fam Issues.

[48] Xue B, McMunn A (2021). Gender differences in unpaid care work and psychological distress in the UK Covid-19 lockdown. PLoS One.

[49] Lai J, Ma S, Wang Y, Cai Z (2020). Factors associated with mental health outcomes among health care workers exposed to coronavirus disease 2019. JAMA Netw Open.

[50] Rossi R, Socci V, Pacitti F (2020). Mental health outcomes among frontline and second-line health care workers during the coronavirus disease 2019 (COVID-19) pandemic in Italy. JAMA Netw Open.

[51] Zhu Z, Xu S, Wang H, Liu Z (2020). COVID-19 in Wuhan: sociodemographic characteristics and hospital support measures associated with the immediate psychological impact on healthcare workers. EClinicalMedicine.

[52] Hassamal S, Dong F, Hassamal S (2021). The psychological impact of COVID-19 on hospital staff. West J Emerg Med.

[53] Li Z, Ge J, Yang M, Feng J (2020). Vicarious traumatization in the general public, members, and non-members of medical teams aiding in COVID-19 control. Brain Behav Immun.

[54] Rostami F, Babaei-Pouya A, Teimori-Boghsani G (2021). Mental workload and job satisfaction in healthcare workers: the moderating role of job control. Front Public Health.

[55] Han PKJ, Strout TD, Gutheil C (2021). How physicians manage medical uncertainty: a qualitative study and conceptual taxonomy. Med Decis Making.

[56] Solis EC, van Hemert AM, Carlier IVE (2021). The 9-year clinical course of depressive and anxiety disorders: new NESDA findings. J Affect Disord.

[57] Lasalvia A, Bonetto C, Porru S (2020). Psychological impact of COVID-19 pandemic on healthcare workers in a highly burdened area of north-east Italy. Epidemiol Psychiatr Sci.

[58] Aburn G, Gott M, Hoare K (2016). What is resilience? An integrative review of the empirical literature. J Adv Nurs.

[59] Di Trani M, Mariani R, Ferri R (2021). From resilience to burnout in healthcare workers during the COVID-19 emergency: the role of the ability to tolerate uncertainty. Front Psychol.

[60] Riehm KE, Brenneke SG, Adams LB (2021). Association between psychological resilience and changes in mental distress during the COVID-19 pandemic. J Affect Disord.

[61] Jiménez-Giménez M, Sánchez-Escribano A, Figuero-Oltra MM (2021). Taking care of those who care: attending psychological needs of health workers in a hospital in Madrid (Spain) during the COVID-19 pandemic. Curr Psychiatry Rep.

[62] Joyce S, Shand F, Tighe J (2018). Road to resilience: a systematic review and meta-analysis of resilience training programmes and interventions. BMJ Open.

[63] Albott CS, Wozniak JR, McGlinch BP (2020). Battle buddies: rapid deployment of a psychological resilience intervention for health care workers during the COVID-19 pandemic. Anesth Analg.

[64] Martin L, McDoall A (2021). The professional resilience of mid-career GPs in the UK: a qualitative study. Br J Gen Pract.

[65] De Simone S, Vargas M, Servillo G (2021). Organizational strategies to reduce physician burnout: a systematic review and meta-analysis. Aging Clin Exp Res.

[66] Pollock A, Campbell P, Cheyne J (2020). Interventions to support the resilience and mental health of frontline health and social care professionals during and after a disease outbreak, epidemic or pandemic: a mixed methods systematic review. Cochrane Database Syst Rev.

